# Testosterone Modulates Virulence-Associated Phenotypes of *Porphyromonas gingivalis* W50

**DOI:** 10.3390/pathogens15090948

**Published:** 2026-09-07

**Authors:** Yvette Amba Kindlund, Rongrong Wu, Kartheyaene Jayaprakash Demirel, Alessandra Neves Guimaraes, Isak Demirel

**Affiliations:** 1 School of Medical Sciences, Örebro University, 701 82 Örebro, Sweden; 2 Department of Oral and Maxillofacial Surgery, Faculty of Medicine and Health, Örebro University, 701 82 Örebro, Sweden; 3 Department of Odontological Research, Public Dental Service, Faculty of Medicine and Health, Örebro University, 701 82 Örebro, Sweden; 4 Department of Periodontology and Implantology, Public Dental Service, Faculty of Medicine and Health, Örebro University, 701 82 Örebro, Sweden

**Keywords:** *Porphyromonas gingivalis*, testosterone, virulence, periodontitis, gingipains, cross-kingdom interaction

## Abstract

*Porphyromonas gingivalis* is a keystone periodontal pathogen strongly associated with periodontitis progression. Although testosterone levels have been associated with periodontal health, its direct effects on *P. gingivalis* virulence remain poorly understood. The aim of this study was to investigate the impact of testosterone exposure on the key virulence characteristics of *P. gingivalis* strain W50. We found that testosterone significantly increased the growth and biofilm biomass of *P. gingivalis* W50 after 48 h. Furthermore, testosterone enhanced the extracellular gingipain activity of both lysine and arginine gingipains from *P. gingivalis*. We also found that IL-1β release from gingival epithelial cells was significantly lowered following infection with testosterone-primed *P. gingivalis* compared with unprimed W50. At the mRNA level, no differences in pro-IL-1β, IL-8, or CXCL10 gene expression were detected in gingival epithelial cells infected with testosterone-primed *P. gingivalis* compared with unprimed W50. Finally, testosterone priming significantly enhanced the ability of *P. gingivalis* to colonize and invade gingival epithelial cells. Overall, these findings indicate that testosterone exposure modulates several virulence-associated phenotypes of *P. gingivalis* W50 in vitro. The mechanisms underlying these effects and their relevance to periodontal disease in vivo remain to be determined.

## 1. Introduction

Periodontitis is a highly prevalent chronic inflammatory disease, with a recent systematic review estimating a prevalence of approximately 62% among dentate adults aged 18 years and older [1], with severe forms contributing substantially to tooth loss and impaired oral health [2]. The disease arises from a dysbiotic shift in the oral microbiota, where an increased abundance of pathogenic bacterial species drives a persistent host inflammatory immune response. This response results in progressive destruction of the tooth-supporting apparatus and may eventually lead to tooth loss [3,4].

Among periodontal pathogens, *Porphyromonas gingivalis* (*P. gingivalis*) is regarded as a keystone pathogen with a central role in promoting microbial dysbiosis and disease progression [5]. *P. gingivalis* expresses several virulence factors such as lipopolysaccharides, gingipains, fimbriae, outer membrane proteins, which enable adhesion, invasion, immune evasion and modulation of host inflammatory responses [6,7,8].

Studies on host–pathogen interactions in periodontitis have mainly focused on how bacterial virulence factors modulate or evade host immune responses [9]. However, recent findings suggest that host-derived factors, such as cytokines and hormones, can directly influence the virulence of bacteria [10,11,12,13]. This supports the concept of cross-kingdom signalling, where bacteria sense host-derived molecules in their microenvironment and adapt their pathogenic potential accordingly. In the periodontal pocket, bacteria are continuously exposed to host-derived molecules present in saliva, serum transudate and gingival crevicular fluid. These molecules may therefore act not only as markers of host physiology, but also as environmental signals that influence bacterial behaviour [14,15,16,17,18]. This concept is particularly relevant in periodontitis, which is a polymicrobial disease with well-established associations to systemic conditions such as cardiovascular disease, metabolic disease and neurodegenerative disorders [19,20].

Current knowledge regarding the direct effects of host mediators, such as sex hormones, on the virulence of periodontal pathogens remains limited. We have previously shown that estradiol increases virulence traits of *P. gingivalis* [12], supporting the concept that sex hormones can directly modulate the pathogenic behaviour of *P. gingivalis*. Previous studies have also shown that *P. gingivalis* can metabolize testosterone, providing further evidence that this bacterium can interact with steroid hormones [21]. However, no specific steroid-binding receptor or molecular sensing mechanism has, to our knowledge, been identified in *P. gingivalis*. In contrast to estradiol, the direct effects of testosterone on periodontal pathogens remain poorly understood. Physiological testosterone levels have been associated with maintaining periodontal homeostasis [22,23,24]. However, both reduced testosterone levels, such as hypogonadism, and supraphysiological exposure, such as anabolic androgenic steroid use, have been linked to adverse periodontal outcomes [25,26,27]. Moreover, we have recently shown that testosterone increases the virulence traits of a uropathogenic *Escherichia coli* [28], suggesting that androgenic signals may influence the pathogenic behaviour of Gram-negative bacteria. However, whether testosterone directly modifies virulence-associated phenotypes of *P. gingivalis*, and the mechanisms underlying such a response, remains unknown.

Understanding how testosterone influences *P. gingivalis* virulence may provide new insight into host–pathogen interaction in periodontitis and identify mechanisms relevant to disease susceptibility and progression. Therefore, the aim of this study was to determine whether testosterone has a direct effect on key virulence traits of *P. gingivalis*.

## 2. Materials and Methods

### 2.1. Bacterial Culture

The *P. gingivalis* W50 strain, provided by Professor M. A. Curtis (Molecular Pathogenesis Group, Queen Mary University of London, London, UK), was used throughout the study. The bacteria were cultured anaerobically at 37 °C in an atmosphere containing 85% N_2_, 5% CO_2_, and 10% H_2_ using an anaerobic chamber (Concept 400-M Anaerobic Workstation; Ruskinn Technology Ltd., Leeds, UK). Cultures were grown in tryptic soy broth (TSB) supplemented with yeast extract (1 mg/mL), hemin (5 μg/mL), and menadione (1 μg/mL). After 48 h of growth, the bacteria were harvested by centrifugation at 10,000 rpm for 10 min, washed, and resuspended in phosphate-buffered saline (PBS). The W50 strain was selected because it is a well-characterized *P. gingivalis* strain commonly used in studies of virulence-associated phenotypes and host–pathogen interactions.

### 2.2. Bacterial Growth Assessment

*P. gingivalis* W50 (5 × 10^6^ CFU/mL) was cultured in TSB supplemented with yeast extract (1 mg/mL), hemin (5 μg/mL), and menadione (1 μg/mL), either in the presence or absence of testosterone (Merck, Darmstadt, Germany). Testosterone was dissolved in DMSO at a stock concentration of 10 mg/mL and stored at −20 °C. The final concentration of DMSO in the bacterial cultures did not exceed 0.0005%, corresponding to the highest testosterone concentration (50 ng/mL). A vehicle control containing 0.0005% DMSO without testosterone was included to control for potential effects of the solvent. Testosterone was tested at the following concentrations: 1 pg/mL, 5 pg/mL, 50 pg/mL, 100 pg/mL, 500 pg/mL, 1 ng/mL, 2 ng/mL, 10 ng/mL, and 50 ng/mL. Cultures were prepared in a 96-well plate with each experimental condition analysed in technical triplicate within the same plate and incubated anaerobically at 37 °C. Optical density at 600 nm was measured using a spectrophotometer (Cytation 3, BioTek Inc., Winooski, VT, USA) after 24 h and 48 h as previously described [10,11,12].

### 2.3. Biofilm Measurement

Following measurement of bacterial growth, attached biofilm biomass was quantified in the same 96-well plates using crystal violet staining. Culture medium was removed, and the wells were rinsed three times with sterile reverse osmosis (RO) water. Biofilms were stained with 0.1% crystal violet (Thermo Fisher Scientific, Waltham, MA, USA) for 20 min, followed by washing with RO water and overnight drying. Bound crystal violet was subsequently solubilized with 95% ethanol, and absorbance was determined at 540 nm using a Cytation 3 spectrophotometer, as previously described [12].

### 2.4. Gingipain Activity Analysis

Following assessment of bacterial growth, culture supernatants were collected from the 96-well plates and clarified by centrifugation at 10,000 rpm for 10 min. The resulting bacteria-free supernatants were transferred to fresh 96-well plates and incubated with either 100 µM Z-His-Glu-Lys-AMC, a substrate for lysine gingipain, or Boc-Phe-Ser-Arg-AMC, a substrate for arginine gingipain (PeptaNova GmbH, Sandhausen, Germany). After incubation at 37 °C for 1 h, extracellular gingipain activity was quantified by measuring fluorescence at 340/440 nm using a Cytation 3 spectrophotometer, as previously described) [12].

### 2.5. Cell Culture

The human gingival squamous cell carcinoma-derived cell line Ca9-22, (GECs) (Tebu-bio, Paris, France) was maintained in Dulbecco’s Modified Eagle Medium (DMEM; Thermo Fisher Scientific, Waltham, MA, USA) supplemented with 10% foetal bovine serum (FBS), 2 mM L-glutamine, and 1 mM non-essential amino acids (Thermo Fisher Scientific, Waltham, MA, USA). The GECs were maintained at 37 °C in a humidified atmosphere containing 5% CO_2_. During experimental procedures, the culture medium was replaced with DMEM containing 2% FBS, 1 mM non-essential amino acids, and 2 mM L-glutamine, as previously described [12].

### 2.6. Cytokine Release

*P. gingivalis* W50 was exposed to testosterone at 5 pg/mL, 100 pg/mL, or 50 ng/mL for 48 h under anaerobic conditions at 37 °C. The lower concentrations were selected to reflect reported salivary testosterone levels, whereas 50 ng/mL represented a high, supraphysiological exposure. After hormone exposure, bacteria were washed twice with PBS to remove residual testosterone and used to infect GECs at an MOI of 100 for 24 h at 37 °C and 5% CO_2_. Culture supernatants were then harvested, clarified by centrifugation at 10,000 rpm for 5 min, and stored at −80 °C until analysis. IL-1β, IL-8, and CXCL10 concentrations were quantified using ELISA MAX Deluxe Sets (BioLegend, San Diego, CA, USA) according to the manufacturer’s instructions.

### 2.7. RNA Isolation, cDNA Synthesis and Quantitative Polymerase Chain Reaction

*P. gingivalis* W50 was exposed to testosterone for 48 h under anaerobic conditions, as described above. After exposure, bacteria were washed twice with PBS to remove residual testosterone and subsequently used to infect GECs at an MOI of 100 for 24 h at 37 °C and 5% CO_2_. Total RNA was isolated using the E.Z.N.A.^®^ Total RNA Kit I (Omega Bio-tek, Inc., Norcross, GA, USA) according to the manufacturer’s instructions. RNA concentration and purity were assessed using a NanoDrop 2000 spectrophotometer (Thermo Fisher Scientific, Waltham, MA, USA). For complementary DNA (cDNA) synthesis, 100 ng of total RNA was reverse-transcribed using the High-Capacity cDNA Reverse Transcription Kit (Applied Biosystems, CA, USA). Quantitative real-time PCR (RT-qPCR) was performed using 5 ng of cDNA and 250 nM of each primer (Table 1) (Eurofins MWG Synthesis GmbH, Ebersberg, Munich, Germany) together with Maxima SYBR Green qPCR Master Mix (Thermo Fisher Scientific). Amplification was carried out for 40 cycles under the following conditions: denaturation at 95 °C for 15 s, annealing at 60 °C for 30 s, and extension at 72 °C for 30 s, using a CFX96 Touch™ Real-Time PCR Detection System (Bio-Rad, CA, USA). A melting curve analysis from 60 °C to 95 °C was performed following amplification. Cycle threshold (Ct) values were obtained, and relative gene expression was calculated using the ΔΔCt method (2^−ΔΔCt^). Expression levels were normalized to the endogenous control GAPDH, as previously described [11,29].

### 2.8. Bacterial Colonization Assay

*P. gingivalis* W50 was cultured anaerobically at 37 °C for 48 h in the presence or absence of testosterone at concentrations of 5 pg/mL, 100 pg/mL, and 50 ng/mL. Following incubation, testosterone was removed by washing the bacteria twice with PBS. Following testosterone priming and washing, bacterial suspensions were adjusted to the same OD600 before infection to standardize the bacterial inoculum and MOI between experimental groups. The bacteria were subsequently labelled with fluorescein isothiocyanate (FITC; Sigma-Aldrich, St. Louis, MO, USA). GECs were then infected with the bacteria at a multiplicity of infection (MOI) of 500 for 3 h at 37 °C in 5% CO_2_, to assess bacterial colonization (including both adherent and intracellular bacteria). After incubation, the cells were washed with PBS, and the FITC-labelled *P. gingivalis* W50 was quantified using the Cytation 3 plate reader. Colonization was expressed as mean fluorescence intensity (MFI), as previously described [12].

### 2.9. Bacterial Invasion Assay

*P. gingivalis* W50 was exposed to testosterone (5 pg/mL, 100 pg/mL, or 50 ng/mL) for 48 h under anaerobic conditions at 37 °C. Following incubation, testosterone was removed by washing the bacteria twice with PBS. Following testosterone priming and washing, bacterial suspensions were adjusted to the same OD600 before infection to standardize the bacterial inoculum and MOI between experimental groups. GECs were then infected with the bacteria at a multiplicity of infection (MOI) of 500 for 2 h at 37 °C in 5% CO_2_. Cells were then washed with PBS and incubated for 1 h in DMEM containing 2% FBS, 300 μg/mL gentamicin, and 200 μg/mL metronidazole to eliminate extracellular bacteria. Following antibiotic treatment, the cells were washed and lysed with 0.1% Triton X-100 in PBS containing 100 mg/L calcium chloride and 100 mg/L magnesium chloride for 10 min under gentle rotation. Cell lysates were plated on TSB agar supplemented with yeast extract (1 mg/mL), hemin (5 μg/mL), and menadione (1 μg/mL). Plates were incubated anaerobically at 37 °C for 48 h, after which viable intracellular bacteria were quantified by colony counting, as previously described [12].

### 2.10. Statistics

All data were analysed with GraphPad Prism 9.0. Statistical differences between groups were analysed using Student’s unpaired *t*-test or one-way ANOVA followed by Bonferroni multiple testing correction. A *p*-value of <0.05 was considered statistically significant. *n* represents the number of independent biological experiments. Results are presented as mean ± standard error of the mean (SEM).

## 3. Results

### 3.1. Testosterone Promotes Increased Growth of P. gingivalis

**Figure 1:**
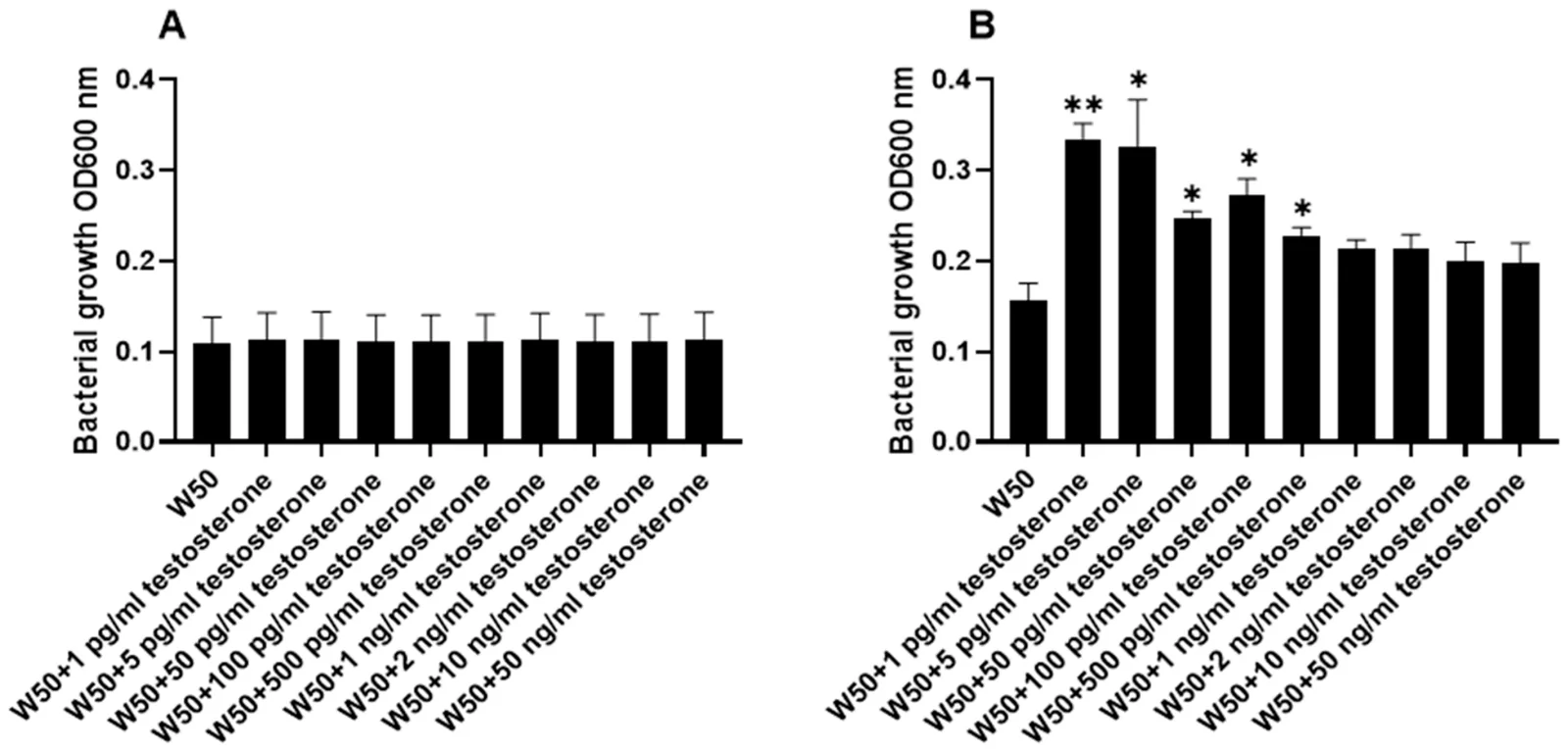
Testosterone alters *P. gingivalis* growth. *P. gingivalis* W50 growth with or without the presence of testosterone (1 pg/mL, 5 pg/mL, 50 pg/mL, 100 pg/mL, 500 pg/mL, 1 ng/mL, 2 ng/mL, 10 ng/mL and 50 ng/mL) after 24 h (**A**) and 48 h (**B**). Data are presented as mean ± SEM of n = 3 independent experiments. Statistical differences between groups were analysed using Student’s unpaired *t*-test. The asterisk distinguishes statistical significance: * *p* < 0.05, ** *p* < 0.01 vs. unstimulated *P. gingivalis* W50.

The results demonstrated no significant increase in W50 growth after 24 h compared to unstimulated W50 (Figure 1A). In contrast, at 48 h, significantly increased W50 growth was observed following exposure to 1 pg/mL, 5 pg/mL, 50 pg/mL, 100 pg/mL, and 500 pg/mL of testosterone compared to unstimulated W50 (Figure 1B). The significant effects were predominantly observed at the lower testosterone concentrations, indicating a non-monotonic concentration–response pattern.

### 3.2. Testosterone Alters P. gingivalis Biofilm Biomass

**Figure 2:**
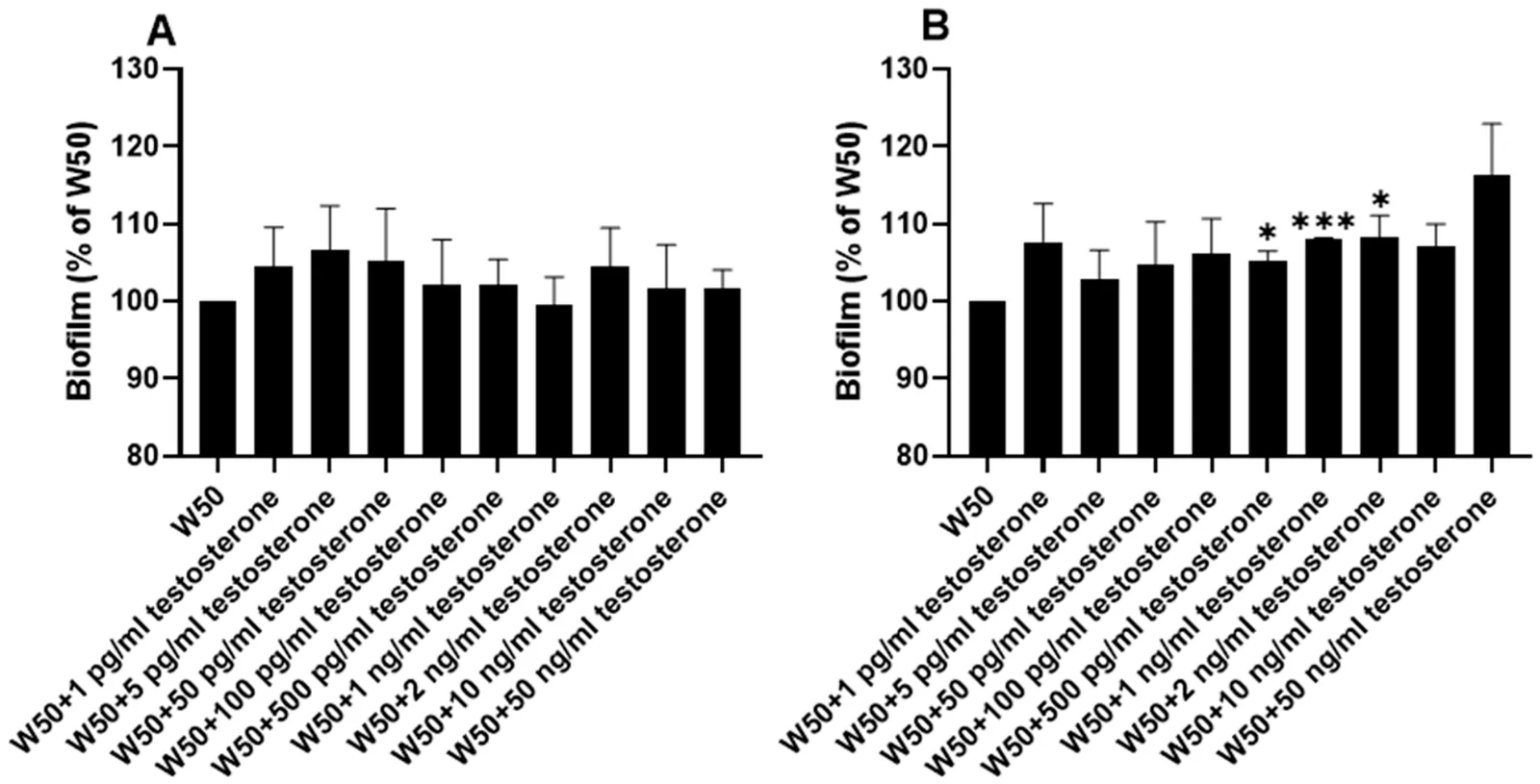
Testosterone alters *P. gingivalis* biofilm biomass. *P. gingivalis* W50 biofilm formation with or without the presence of testosterone (1 pg/mL, 5 pg/mL, 50 pg/mL, 100 pg/mL, 500 pg/mL, 1 ng/mL, 2 ng/mL, 10 ng/mL and 50 ng/mL) after 24 h (**A**) and 48 h (**B**). Data are presented as % of W50, mean ± SEM of n = 3 independent experiments. Statistical differences between groups were analysed using Student’s unpaired *t*-test. The asterisk distinguishes statistical significance: * *p* < 0.05, *** *p* < 0.001 vs. unstimulated *P. gingivalis* W50.

Next, we further investigated the effects of testosterone on *P. gingivalis* by assessing biofilm biomass. The results demonstrated no significant increase in biofilm biomass after 24 h compared to unstimulated W50 (Figure 2A). In contrast, after 48 h, modest but statistically significant increases in biofilm biomass of 5%, 8%, and 8% were observed at 500 pg/mL, 1 ng/mL, and 2 ng/mL testosterone, respectively, compared with unstimulated W50 (Figure 2B).

### 3.3. Testosterone Increases P. gingivalis Extracellular Gingipain Activity

**Figure 3:**
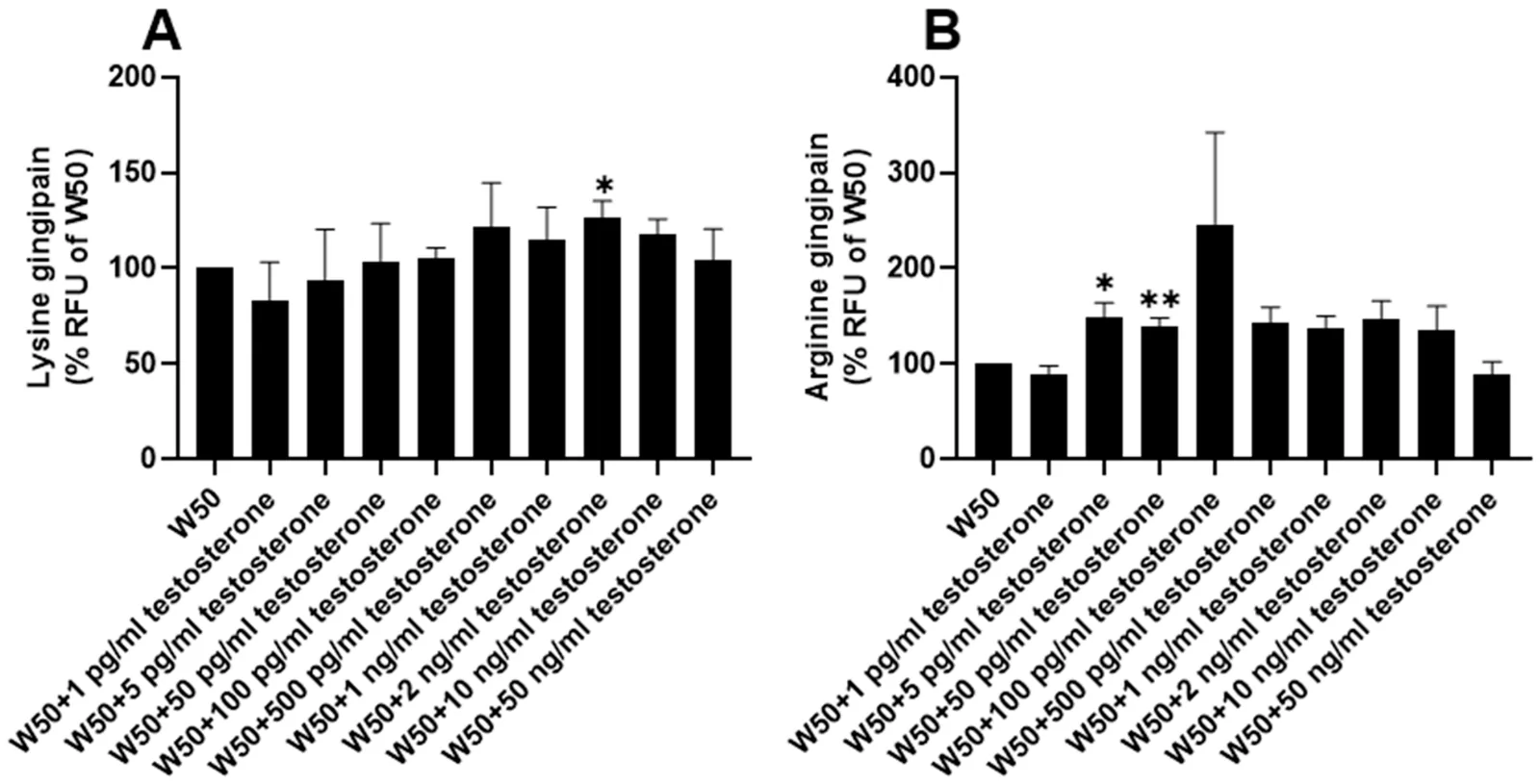
Testosterone increases *P. gingivalis* extracellular gingipain activity. *P. gingivalis* W50 lysine (**A**) and arginine (**B**) gingipain activity assay with or without the presence of testosterone (1 pg/mL, 5 pg/mL, 50 pg/mL, 100 pg/mL, 500 pg/mL, 1 ng/mL, 2 ng/mL, 10 ng/mL and 50 ng/mL) during 48 h (**A**,**B**). Data are presented as % relative fluorescence units (RFU) of W50 and mean ± SEM of n = 3 independent experiments. Statistical differences between groups were analysed using Student’s unpaired *t*-test. The asterisk distinguishes statistical significance: * *p* < 0.05, ** *p* < 0.01 vs. unstimulated *P. gingivalis* W50.

To further investigate the effects of testosterone on virulence-associated traits of *P. gingivalis*, extracellular gingipain activity was analysed. As shown in Figure 3A, lysine gingipain activity after 48 h was significantly increased by 26% following exposure to 2 ng/mL testosterone compared with unstimulated W50. In addition, arginine gingipain activity was significantly increased by 50% and 40% following exposure to 5 pg/mL and 50 pg/mL testosterone, respectively, compared with unstimulated W50 (Figure 3B).

### 3.4. Testosterone Altered IL-1β Release from Gingival Epithelial Cells

**Figure 4:**
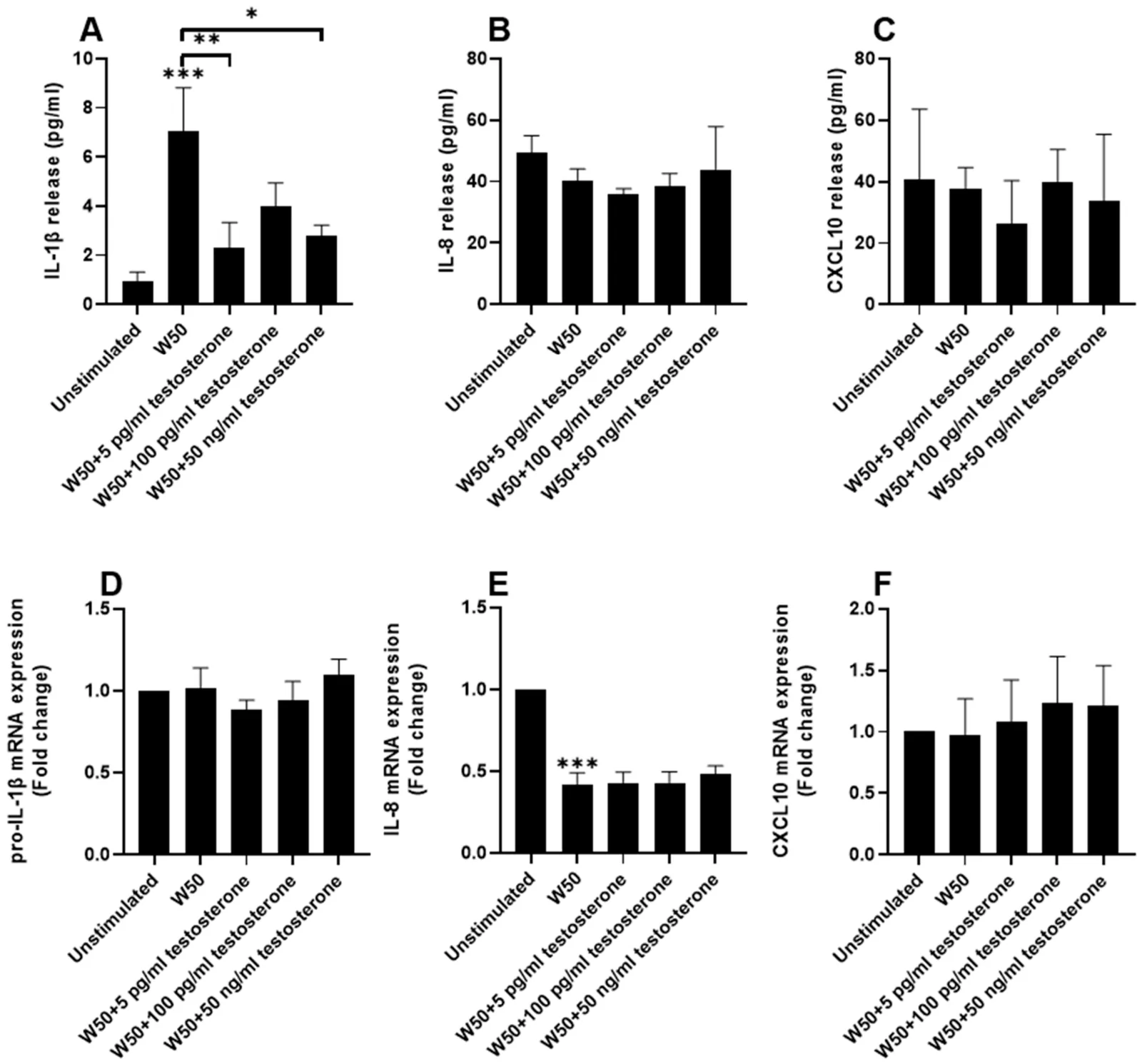
Testosterone alters *P. gingivalis*-induced cytokine release and inflammatory gene expression in gingival epithelial cells. Release of IL-1β (**A**), IL-8 (**B**) and CXCL10 (**C**) from GECs infected with *P. gingivalis* W50 with or without testosterone-priming (5 pg/mL, 100 pg/mL, and 50 ng/mL) for 24 h. Real-time qPCR analysis of pro-IL-1β (**D**), IL-8 (**E**), and CXCL10 (**F**) gene expression in GECs infected with *P. gingivalis* W50 with or without testosterone-priming (5 pg/mL, 100 pg/mL, and 50 ng/mL) for 24 h. Data are presented as mean ± SEM of n = 4 independent experiments. Statistical differences between groups were analysed using one-way ANOVA followed by Bonferroni multiple testing correction. The asterisk distinguishes statistical significance: * *p* < 0.05, ** *p* < 0.01, *** *p* < 0.001 vs. unstimulated GECs.

Next, we investigated whether testosterone-primed *P. gingivalis* could alter the release of the cytokines IL-1β, IL-8, CXCL10 from gingival epithelial cells. We found a significant increase in IL-1β release after infection with unprimed W50 compared to unstimulated GECs (Figure 4A). Furthermore, we also found significantly lower IL-1β release from GECs infected with testosterone-primed W50 compared to GECs infected with unprimed W50 (Figure 4A). However, we did not find any differences in IL-8 (Figure 4B) or CXCL10 (Figure 4C) release from GECs infected with primed or unprimed W50.

### 3.5. P. gingivalis Alters Induced IL-8 Gene Expression in Gingival Epithelial Cells

Real-time qPCR analysis revealed no significant change in pro–IL-1β (Figure 4D) or CXCL10 (Figure 4F) gene expression in W50 infected GECs compared to unstimulated cells. However, IL-8 gene expression was significantly reduced after W50 infection compared to unstimulated cells (Figure 4E). In contrast, no differences in *pro–IL-1β*, *IL-8*, or *CXCL10* gene expression were observed in GECs infected with testosterone-primed W50 compared with unprimed W50 (Figure 4D–F).

### 3.6. Testosterone Facilitates Increased Colonization and Invasion of Gingival Epithelial Cells

**Figure 5:**
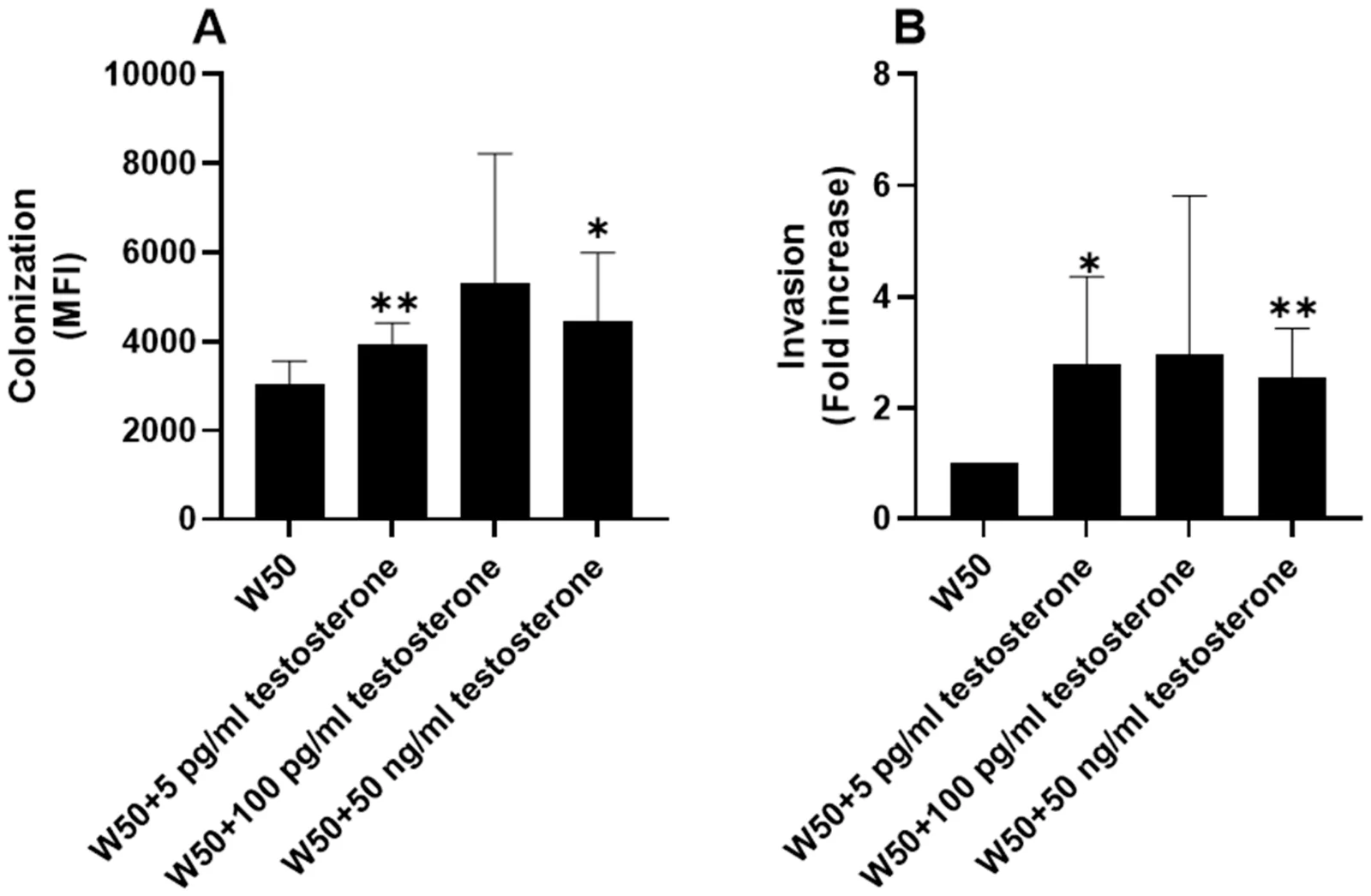
Testosterone mediated increased colonization and invasion. *P. gingivalis* W50 colonization (**A**) or invasion (**B**) of gingival epithelial cells infected with *P. gingivalis* with or without testosterone-priming (5 pg/mL, 100 pg/mL, and 50 ng/mL) after 3 h (**A**) or 2 h (**B**). Colonization quantification of FITC-labelled *P. gingivalis* W50 is presented as mean fluorescence intensity (MFI). Invasion is presented as fold change relative to unprimed W50. Data are presented as mean ± SEM of n = 7 (**A**) or 5 (**B**) independent experiments. Statistical differences between groups were analysed using Student’s unpaired *t*-test. The asterisk distinguishes statistical significance: * *p* < 0.05, ** *p* < 0.01 vs. unstimulated *P. gingivalis* W50.

We proceeded to evaluate the effects of testosterone on the ability of *P. gingivalis* to colonize and invade GECs. Colonization of GECs appeared to increase following testosterone priming of *P. gingivalis* W50 compared with unprimed W50. This increase was significant for W50 primed with 5 pg/mL and 50 ng/mL of testosterone (Figure 5A). Furthermore, testosterone priming enhanced the invasive capacity of *P. gingivalis* W50, with invasion increasing 2.8-fold and 2.5-fold following priming with 5 pg/mL and 50 ng/mL testosterone, respectively, compared with unprimed W50 (Figure 5B).

## 4. Discussion

A deeper understanding of host–pathogen interactions in periodontal disease is essential for clarifying disease mechanisms and identifying new therapeutic strategies. While periodontal pathology is primarily associated with dysbiotic bacterial communities and dysregulated host inflammatory responses, it is becoming increasingly clear that host-derived factors can also directly influence bacterial behaviour. Changes in systemic testosterone levels have been associated with altered immune responses and increased susceptibility to disease [30,31]. In the periodontal context, physiological testosterone levels have been associated with maintaining periodontal homeostasis [22,23,24], whereas both reduced testosterone levels, such as hypogonadism, and supraphysiological exposure, such as anabolic androgenic steroid use, have been linked to adverse periodontal outcomes [25,26,27]. In line with this broader concept, our previous work showed that testosterone increases virulence traits of uropathogenic *Escherichia coli*, suggesting that androgenic signals may influence the pathogenic behaviour of Gram-negative bacteria [28]. However, the direct effects of testosterone on *P. gingivalis* remain poorly understood. The present study demonstrates that testosterone can directly enhance several virulence-associated traits of *P. gingivalis*, including bacterial growth, biofilm biomass, extracellular gingipain activity, and colonization and invasion of GECs, while also modulating epithelial immune responses.

Salivary testosterone concentrations vary between men and women and are influenced by age, circadian rhythm, and physiological status [32,33,34,35]. Reported salivary testosterone levels are approximately 1.6–14.1 pg/mL in women and 23.5–108.3 pg/mL in men in morning saliva [32,36], supporting the relevance of investigating low testosterone concentrations in relation to bacterial behaviour. A key observation in the present study was that testosterone did not significantly affect *P. gingivalis* growth after 24 h, whereas bacterial growth was significantly increased after 48 h, particularly at lower testosterone concentrations. This time-dependent effect suggests that testosterone may not primarily induce immediate bacterial proliferation but rather influence bacterial adaptation over time. The stronger response observed at the lower testosterone concentrations may therefore reflect a physiologically relevant bacterial sensitivity to subtle endocrine changes within the periodontal microenvironment. Importantly, testosterone did not produce a consistent concentration-dependent response across all endpoints, and the highest concentrations frequently showed no significant effect. This non-monotonic pattern suggests that testosterone modulates specific virulence-associated phenotypes rather than uniformly increasing *P. gingivalis* virulence.

In addition to bacterial growth, testosterone significantly increased biofilm biomass after 48 h, while only a non-significant increasing trend was observed after 24 h. Because bacterial growth was also increased after 48 h, the higher biofilm biomass may partly reflect an increased bacterial population rather than an increased biofilm-forming capacity per bacterium. Accordingly, these findings should be interpreted as an increase in total attached biofilm biomass following testosterone exposure. Nevertheless, an increase in total biofilm biomass remains biologically relevant, as it may contribute to greater bacterial persistence within the periodontal environment. We have previously reported similar hormone-associated patterns for estradiol-primed *P. gingivalis* [12] and testosterone-primed uropathogenic *Escherichia coli* [28]. This pattern is also in line with previous findings showing that sex hormones can modulate biofilm formation in Gram-negative bacteria. Al-Zawity et al. demonstrated that estradiol increased biofilm biomass and remodelled biofilm structure in *Pseudomonas aeruginosa* [13]. Biofilm formation is of major clinical relevance in periodontal disease, as biofilms enable bacterial persistence, protection from host immune responses, and increased tolerance to conventional antimicrobial agents, thereby contributing to chronic infection and reduced treatment efficacy [37,38].

Furthermore, we found that testosterone increased extracellular lysine and arginine gingipain activity in *P. gingivalis* cultures. Gingipains are central virulence factors of *P. gingivalis* and contribute to nutrient acquisition, tissue destruction, immune evasion, cytokine modulation, adherence, colonization, invasion and systemic dissemination [38,39,40,41,42,43,44]. Because bacterial growth was also increased after 48 h, the higher extracellular gingipain activity may partly reflect an increased bacterial population. Accordingly, these findings should be interpreted as an increase in total extracellular gingipain activity following testosterone exposure rather than increased gingipain activity per bacterium. This observation is consistent with the broader concept that host-derived factors can influence bacterial enzymatic activity and pathogenic behaviour [11,45]. Taken together, the increased extracellular gingipain activity may strengthen key virulence mechanisms of *P. gingivalis*.

We also investigated the effects of testosterone priming on *P. gingivalis* colonization and invasion of gingival epithelial cells. We found that testosterone priming enhanced the ability of *P. gingivalis* to colonize and invade GECs. These mechanisms are highly relevant for *P. gingivalis* pathogenicity, as attachment to gingival epithelial cells represents an important early step in oral tissue colonization, while invasion may facilitate intracellular persistence and evasion of extracellular immune defences [46,47,48]. Fimbrial and non-fimbrial adhesins, together with gingipains, contribute directly or indirectly to epithelial adherence and invasion. Arginine gingipains are important for fimbrial maturation, whereas lysine gingipain has been shown to contribute to adherence to oral epithelial cells [7,9,39]. Together, these findings suggest that testosterone may promote epithelial interaction by enhancing gingipain activity, biofilm-associated adaptation, and by the adhesive and invasive capacity of *P. gingivalis*, although the precise mechanisms remain to be elucidated.

We continued to investigate whether testosterone-primed *P. gingivalis* could alter the release of cytokines from GECs. We found that *P. gingivalis* induced increased IL-1β release, but not IL-8 or CXCL10, and that this release was lowered by testosterone-primed *P. gingivalis*. At the mRNA level, no differences in *pro-IL-1β*, *IL-8*, or *CXCL10* gene expression were observed in GECs infected with testosterone-primed *P. gingivalis*. The reduced extracellular IL-1β concentration may reflect altered cytokine processing or secretion, but may also result from increased proteolytic degradation by bacterial gingipains. This is particularly relevant given the increased extracellular gingipain activity observed following testosterone exposure and previous studies showing that *P. gingivalis* gingipains can degrade or modulate cytokines such as IL-8 and IL-1β [42,49,50]. The unchanged *pro-IL-1β* mRNA expression does not distinguish between these possibilities. As inflammasome activation and downstream processing of IL-1β [51,52] were not directly assessed in the present study, the mechanisms underlying the reduced extracellular IL-1β levels remain to be determined. Taken together, these findings indicate that testosterone exposure alters the interaction between *P. gingivalis* and epithelial inflammatory responses. At this stage, the reduced extracellular IL-1β level should be regarded as a phenotypic observation rather than evidence of a specific underlying mechanism. Further mechanistic studies assessing IL-1β processing, inflammasome activation, and gingipain-mediated cytokine degradation will be required to determine the pathway responsible for this effect.

Together with previous findings showing that estradiol enhances *P. gingivalis* virulence traits, including growth, biofilm formation, gingipain activity, and immune modulation [12], the present results support the concept that sex hormones can directly shape bacterial behaviour in the periodontal microenvironment. Given known associations between hormonal fluctuations and periodontal status, such as during pregnancy and menopause [53,54], the present findings extend this concept to testosterone and highlight its potential role in disease modulation. However, it remains unclear whether testosterone is sensed through a specific bacterial receptor, membrane-associated sensor, or indirect metabolic adaptation. Identifying such mechanisms will be important for understanding how host micromilieu regulates *P. gingivalis* virulence and whether this process can be therapeutically targeted.

This study has several limitations that should be considered when interpreting the findings. Only the *P. gingivalis* W50 strain was investigated, and the use of the carcinoma-derived Ca9-22 cell line may limit generalizability to other bacterial strains and primary gingival epithelial cells. Furthermore, the single-species biofilm model used here does not fully reflect the complexity of polymicrobial periodontal biofilms and their interspecies interactions [55]. Some experiments were based on a limited number of biological replicates, and the molecular mechanism by which *P. gingivalis* responds to testosterone was not investigated. In addition, the increased bacterial growth may have contributed to the higher biofilm biomass and extracellular gingipain activity. Further studies using additional strains and more physiologically relevant models will be needed to confirm these findings and clarify the underlying mechanisms.

## 5. Conclusions

In conclusion, the present study shows that testosterone exposure modulates several virulence-associated phenotypes of *P. gingivalis* W50 in vitro, including bacterial growth, biofilm biomass, extracellular gingipain activity, and epithelial colonization and invasion, while also affecting epithelial IL-1β responses. These findings support a role for testosterone as a host-derived factor capable of influencing *P. gingivalis* behaviour. However, the underlying mechanisms and the in vivo relevance of these observations remain to be determined.

## Figures and Tables

**Table 1:** Primers used for quantitative real time PCR.

Gene Symbol	Oligonucleotide Sequences (5′–3′)
*pro-IL-1β*	F: CCACAGACCTTCCAGGAGAATG R: GTGCAGTTCAGTGATCGTACAGG
*IL-8*	F: GAGAGTGATTGAGAGTGGACCAC R: CACAACCCTCTGCACCCAGTTT
*CXCL10*	F: GGTGAGAAGAGATGTCTGAATCC R: GTCCATCCTTGGAAGCACTGCA
*GAPDH*	F: GTCTCCTCTGACTTCAACAGCG R: ACCACCCTGTTGCTGTAGCCAA

## Data Availability

All data generated or analysed during this study are included in this published article.
